# Selective androgen receptor modulators: a critical appraisal

**DOI:** 10.3389/fendo.2025.1634799

**Published:** 2025-09-26

**Authors:** Peter Bond, Diederik L. Smit, Tijs Verdegaal, Willem de Ronde

**Affiliations:** ^1^ Android Health Clinic, Department of Performance and Image-enhancing Drugs Research, Utrecht, Netherlands; ^2^ Department of Internal Medicine, Zaandam Medical Centre, Zaandam, Netherlands; ^3^ Department of Internal Medicine, Spaarne Gasthuis, Haarlem, Netherlands

**Keywords:** selective androgen receptor modulators, SARMs, androgens, androgen pharmacology, androgen receptor

## Abstract

The concept of selective androgen receptor modulators (SARMs) was introduced in 1999 in analogy to selective estrogen receptor modulators (SERMs). The primary goal was to separate the unwanted androgenic or virilizing effects from the anabolic or myotrophic effects. This separation would result from tissue-selective effects. In this paper, we critically appraise the evidence behind SARMs’ purported tissue selectivity with emphasis on historical androgen research, which, in essence, tried to achieve the same goal by modifying the steroid nucleus. While SARMs demonstrate favorable ‘anabolic-androgenic ratios’ in preclinical studies, much of this apparent selectivity may stem from a lack of steroidal metabolism — such as 5α-reduction and 3α/β-reduction — when compared with steroidal androgens that are susceptible to these metabolic pathways. Emerging evidence suggests that differential recruitment of coregulators and differences in activation of nongenomic signaling pathways may contribute to tissue-selective effects, but it remains unclear whether this translates to clinically meaningful tissue selectivity. Clinical trials reveal some efficacy of SARMs in terms of improvements in body composition or anti-tumor activity in advanced breast cancer, yet these results might equally well have been achieved with conventional androgens as head-to-head trials are lacking. Furthermore, the absence of estrogenic activity poses a clinical challenge, especially regarding bone health and sexual function in men. Overall, while SARMs present an attractive therapeutic concept, robust evidence supporting their superiority over traditional androgens remains incomplete, warranting cautious interpretation and further comparative research.

## Introduction

The identification, isolation (1) and synthesis (2) of testosterone in 1935 marked the inception of the field of modern androgen research. Testosterone’s steroid nucleus — consisting of three cyclohexane rings and one cyclopentane ring joined together — formed the molecular skeleton for organic chemists to tinker with in order to gear its physiologic effects in a desired direction. Separating the androgenic or virilizing effects from the anabolic or myotrophic effects was — and is — of special interest, especially for application in muscle-wasting conditions, particularly in children and women. A tremendous effort was made in pursuit of a ‘pure’ anabolic steroid. Thousands of chemical configurations were tried and tested in the decades following testosterone’s discovery. Some 650 of these derivatives of testosterone are documented in the seminal work *Androgens and Anabolic Agents* by the late Julius A. Vida, a prominent Hungarian-American chemist (3). Much of the work relied on experimental animal data that yielded ‘anabolic-androgenic ratios’. While never formally standardized, the 1953 Hershberger assay — a modification of a bioassay described by Eisenberg and Gordan three years earlier (4) — became the *de facto* standard for assessing anabolic versus androgenic effects of androgenic compounds of interest (5). The anabolic effect was derived from changes in the levator ani weight and the androgenic effect from changes in the ventral prostate or seminal vesicle weight in castrated male rats following androgen administration. This approach yielded a wide array of compounds of interest with promising ratios — whether reflecting a true dissociation or not — yet none of them was void of virilizing effects.

Reports from the early 1970s of multiple androgen receptors (ARs) with distinct anabolic and androgenic activities provided a mechanistic underpinning for the observed favorable ratios and offered hope for further clinical development as compounds with differential receptor affinities might exploit this finding (6, 7). Two decades later, an N-terminal truncated form of the full-length AR was identified (8). However, rigorous experiments with various AR ligands failed to demonstrate differences in the activities between these two AR isoforms (9, 10). For completeness, it is worth noting that several AR splice variants (e.g. AR-V7) have been identified in cancers (in particular breast and prostate cancer), although most lack the ligand-binding domain (11). In retrospect, the earlier discovery of 5α-reductase, a family of three isozymes encoded by *SRD5A1*, *SRDA5A2* and *SRD5A3*, and its role in amplifying testosterone’s effects in target tissues via conversion into the more potent androgen 5α-dihydrotestosterone (12–14) was arguably the most significant mechanistic insight into how tissue-specific androgenic potency can rise separate from anabolic effects. This ultimately led to the development and market approval of 5α-reductase inhibitors, which have been in use since the 1990s to treat two androgen-driven processes: benign prostate hyperplasia (15) and androgenetic alopecia (16).

Despite the impressive collective effort behind the similarly astonishing number of evaluated compounds, no clear separation of androgenic and anabolic effects was achieved bar from the lack of metabolic amplification in tissues expressing 5α-reductase. In analogy to selective estrogen receptor modulators (SERMs), Negro-Vilar introduced the concept of selective androgen receptor modulators (SARMs) in his pioneering paper published in 1999 (17). He highlighted the clinical limitations of steroidal androgens, recent advances in AR structure and function, and described the desired activity profiles of a SARM for various medical indications. An example of a desired activity profile for male hypogonadism proposed by him is shown in **Table 1**. The essence of SARMs largely overlaps with the quest of organic chemists decades earlier to modify the steroid nucleus to absolve side effects while retaining anabolic actions. The concept of SARMs, however, is determined to distinguish itself from this past failed quest through application of modern knowledge and techniques, and liberation from the chemical constraints imposed by steroid-based structures by pivoting to nonsteroidal molecules such as quinolinones, arylpropionamides, indole derivatives, and others. Their tissue selectivity, which should minimize side effects and optimize the desired action, is proposed to follow from differential tissue- or cell-specific combinatorial control of transcriptional coregulators. Consequently, SARMs are often touted to have less side effects than testosterone or other conventional steroidal androgens (18–20), yet strong supporting evidence is lacking. SARMs have therefore drawn criticism, being dubbed a misleading marketing term rather than an accurate pharmacological description (21).

**Table 1:** Desired profile of activity of a SARM for male hypogonadism as proposed by Negro-Vilar (17).

Tissue/parameter	Effect
Prostate/sex accessory tissues	Stimulatory, but less than DHT
Libido	Stimulatory
Inhibition of gonadotropins	Present
Hair growth	Stimulatory
Bone growth	Stimulatory
Muscle mass/strength	Stimulatory
Fat-free mass	Increase
Lipids/cardiovascular risk factors	Neutral
Blood pressure/fluid retention	Neutral
Erythropoiesis	Weakly stimulatory
Liver function (enzyme elevation)	Neutral
Breast (gynecomastia)	Neutral

In this paper we critically evaluate the evidence for the purported enhanced tissue selectivity of SARMs compared with traditional steroidal androgens, and assess their results in clinical research.

## Dissecting the evidence for tissue selectivity

### The Hershberger assay and ‘anabolic-androgenic ratio’

The dissociation of anabolic from androgenic effects is proposed to result from tissue-selective actions. A classic method to quantify these is the aforementioned Hershberger assay (5). In the original experiment, 21-day-old rats are castrated and then receive daily administration of the test androgen for 7 days. On day 8, animals are sacrificed and dissected to measure the weights of the levator ani muscle, ventral prostate and seminal vesicles. The ratio between the levator ani and ventral prostate or seminal vesicles weight changes, compared with castrate controls, is calculated to indicate the compound’s relative anabolic versus androgenic activity. The levator ani weight change serves as a surrogate for anabolic action and that of the ventral prostate for androgenic action. These numbers are then compared with those of a reference standard, usually testosterone or testosterone propionate for injectable and 17α-methyltestosterone for oral compounds. We will discuss this assay because it holds historical value and is applied in contemporary SARM research.

While an appealing method, results of the Hershberger assay should be interpreted with caution because of several limitations. First, the levator ani muscle is doubtful to be representative of skeletal muscle. As early as 1957, researchers questioned its validity as an index of anabolic action, arguing that its growth is sex-linked rather than generally myotrophic (22). Despite its common name (a misnomer for the dorsal bulbocavernosus/bulbospongiosus muscle (23, 24)), contraction of the muscle does not lift the anus, is only present in male rats and active only during copulation (24). As a reproductive muscle, it is exquisitely sensitive and dependent on androgens, atrophying significantly after castration (22) and having a severalfold higher AR content than skeletal muscle (25). Second, while rat prostate size dose-dependently increases in response to androgens, human prostate volume remains unchanged even with supraphysiological dosages up to 600 mg testosterone enanthate weekly (approximately 6 times the dosage used for testosterone replacement therapy) for 20 weeks (26). While differences in lifespan and the rate of biological processes between rats and humans limit comparison, it nevertheless seems that rat prostate weight changes in response to androgens are not translatable to humans. Third, there is no empirical support to assume that size changes of the rat ventral prostate or seminal vesicles reflect clinically desirable tissue selectivity (e.g., the assumption that a favorable response of the ventral prostate to an androgen would also signify a favorable response on erythropoiesis, or any other undesired androgenic effect, in relation to its anabolic effect). Fourth, the apparent dissociation of anabolic from androgenic effects depends on the timing of observation, as tissues respond differently across androgen concentration ranges (27).

Despite these drawbacks, pharmaceutical companies continue to use (modifications of) this assay for SARMs screening (28–37). Compounds with ‘favourable’ ratios are further explored and, conversely, compounds with ‘unfavorable’ ratios are discarded, even though the assay holds questionable clinically translational value beyond demonstrating any androgenic action. The failure of translational success from the hundreds, if not thousands, of steroidal compounds evaluated by this bioassay to translate into clinical utility — despite a substantial fraction demonstrating favorable results — might as well serve as an empirical testimony to abandon it for screening purposes.

### *In vitro* studies and the illusion of enhanced selectivity

More recent investigations complement *in vivo* animal research with *in vitro* transactivation assays. These involve transfection of cell lines with a luciferase reporter gene under transcriptional control of androgen response elements (AREs), allowing androgen activity to be quantified trough luminescence measurements. Ostrowski et al. applied this method in C2C12 mouse myoblasts and rat prostate epithelial cell lines (38). They evaluated various concentrations of the bicyclic hydantoin SARM BMS-564929 to establish the potency (EC_50_, calculated as the concentration at which 50% of the maximum stimulatory effect of dihydrotestosterone [DHT] was achieved) compared with testosterone in either cell line. BMS-564929 had an EC_50_ of 0.44 and 8.66 nM in the myoblasts and prostate cells, respectively, whereas testosterone’s values were 2.81 and 2.17 nM. This suggests a roughly 20-fold selectivity in muscle over prostate cells. The researchers also investigated the androgenic and anabolic effects of BMS-564929 using a model similar to the Hershberger assay. The ED_50_ (defined as the dose at which tissue wet weight reached 50% of the weight of that of the sham group) was 0.0009 mg/kg in the levator ani and 0.14 mg/kg in the prostate, indicating a 160-fold selectivity for the levator ani over the prostate. This result translates to an 80-fold selectivity over testosterone propionate in the same assay. At first glance, these results look remarkably promising. However, as excellently argued by Goa and Dalton (39), these results (and that of many other SARMs) are likely the result of lack of androgenic amplification by 5α-reductase — unlike with testosterone. The dose-response curves of BMS-564929 *vis a vis* testosterone with a 5α-reductase inhibitor from a comparable study (40) show considerable overlap.

Comparison of a SARM with testosterone can thus create the misleading impression of relative tissue selectivity as testosterone’s effects are potentiated via conversion to DHT in certain tissues such as the prostate. A similar problem arises when a comparison is made with DHT (as occasionally done (28, 32, 41)) instead of testosterone. Whereas testosterone’s effect is amplified in tissues exhibiting 5α-reductase activity, DHT is inactivated in tissues with 3α/β-dehydrogenase activity yielding the inactive metabolites 3α- and 3β-androstanediol (42). Importantly, irreversible 3α-reduction of DHT occurs in skeletal muscle (43) — both in rodents (including in the levator ani muscle) (44) and humans (45).

Obviously, part of SARMs’ tissue selectivity stems from differences in metabolic fate compared with steroidal androgens such as testosterone and DHT. However, certain decades-old steroidal androgens also exhibit these metabolic features, e.g. oxandrolone (46). Notably, nandrolone, a testosterone derivative lacking the methyl group at the C19 position, is 5α-reduced to dihydronandrolone (DHN). DHN has lower AR binding affinity than nandrolone and thus may result in reduced rather than amplified activity in tissues expressing 5α-reductase (47). Arguably, such steroidal androgens offer a more meaningful basis for comparison in these assays than testosterone and DHT, although other limitations of these assays remain.

### Lack of estrogenic activity and metabolites: a clinical challenge

Finally, an overarching issue in SARM development for many clinical applications is their lack of estrogenic effects. Unlike some steroidal androgens (in particular testosterone), current SARMs are not aromatized into estrogens and do not exhibit intrinsic estrogenic activity. They therefore cannot replace or supplement estrogen. When SARMs suppress gonadotropin release, testosterone and estradiol levels fall, potentially causing a relative or absolute estrogen deficiency. In men, experimentally induced estrogen deficiency increases body fat and contributes to declines in sexual function (48). Over time, longstanding inadequate estrogen levels may increase the risk of osteoporosis (49, 50). Even when SARMs achieve substantial tissue selectivity, their inability to preserve or supplement estrogenic action presents an additional clinical challenge. Nevertheless, because negative feedback on the hypothalamo-pituitary gonadal axis (HPGA) largely relies on estrogenic feedback, gonadotropin suppression may be mild in therapeutic dosages, as also is the case for nonaromatizable steroidal androgens (51–54).

### Beyond metabolism

Early SERMs, such as tamoxifen, were initially viewed as antiestrogens. Their surreptitious tissue selectivity was unknown at the time and identified only later (55). The molecular basis for this selectivity remained elusive until the discovery of a second estrogen receptor isoform (ERβ; leading to renaming of the classical ER to ERα) in 1996 provided some insight (56, 57). Future developments in the field could exploit this biological ‘gift’ for potential selectivity. As the AR lacks multiple receptor isoforms with distinct biological action, this relatively easy vector to establish tissue selectivity is unavailable in SARM development. Instead, tissue selectivity needs to rely on other mechanisms, such as the interaction with the AR and tissue-specific differences in AR coregulators and their recruitment.

While not purposely engineered for it, the estrogenic action of tamoxifen in the uterus relies on high expression of steroid receptor coactivator 1 (SRC-1) in endometrial cells (58). This highlights the critical role of coregulators that are differentially expressed in tissues and suggests that a similar approach could apply to SARMs (59). The underlying idea is that different ligands induce a different conformational change in the AR ligand-binding pocket (LBP), which, in turn, induces a conformational shape at the AR surface that differentially binds coregulators and thereby exhibits a novel profile of coregulator interaction that favors a desired tissue selectivity. This might also include altered post-translational modifications of the AR, as the receptor contains many phosphorylation, and to a lesser extent acetylation, SUMOylation, ubiquitination and methylation sites across its N-terminal, DNA-binding, hinge, and ligand binding domains (60). SARM research on post-translational modifications remains unexplored, however.

Ostrowski et al. provided structural evidence for the concept of adopting a distinct conformational shape by resolving the cocrystal structure of the AR ligand-binding domain (LBD) bound to the SARM BMS-564929 at 3 Å resolution, and compared this with the known structure of the DHT-bound AR LBD at 2 Å resolution (38). Unlike DHT, BMS-564929 interacted with the side chain of F764 and lacks the hydrogen bond with T877. This forms a unique LBP which likely determines distinct surface features for differential coregulator action. These findings establish credibility for the concept of differential coregulator recruitment, echoing the mechanism of SERMs (61).

Additional support comes from a study by Hikichi et al. (34). By using a mammalian two-hybrid (M2H) assay expressing the AR and 112 cofactors in human embryonic kidney cells (293T), the authors determined the cofactor recruitment profile of the steroidal ‘SARM’ TSAA-291 and compared that with DHT. Of the 112 cofactors, 55 bound to AR, with 12 demonstrating differences in recruitment by TSAA-291 compared with DHT. Among these, the protein inhibitor of activated STAT 1 (PIAS1) was notable for its differential recruitment. The authors further showed that PIAS1 was more highly expressed in prostate tissue than in skeletal muscle, and that PIAS1 functions as a coactivator of AR transcriptional activity. PIAS1 overexpression increased AR transcriptional activity, while silencing of PIAS1 by siRNA reduced DHT-induced prostate-specific antigen (PSA) production. This indicates that PIAS1 plays a functional role in AR signaling and suggest that differential recruitment of cofactors like PIAS1 may contribute to tissue-selective activity of TSAA-291. While suggestive, these findings do not conclusively prove that coregulator differences explain clinically meaningful tissue selectivity.

Importantly, coregulator research followed observations of selective activity in bioassays rather than that it preceded or guided the rational design of SARMs.

Nongenomic pathways may also play a role in SARM tissue selectivity. Narayanan et al. investigated this using the arylpropionamide SARM S-22 (62). *Xenopus* oocyte maturation, a purely nongenomic effect that depends on the AR, was induced by both S-22 and testosterone. Previous research demonstrated that some steroidal androgens, such as DHT and R1881, do not induce *Xenopus* oocyte maturation, whereas others, such as testosterone and androstenedione, do (63). Additionally, Narayanan et al. showed that LNCaP cells treated with S-22 or DHT showed differences in phosphorylation of various kinases. S-22 phosphorylated p38 MAPK, whereas DHT phosphorylated extracellular signal-regulated kinase (ERK). In U2OS osteoblasts transfected with AR, both DHT and S-22 phosphorylated ERK to a similar degree. These findings suggest a shared action on bone but divergent on the prostate resulting from differences in nongenomic signaling pathways. Importantly, modulation of these pathways affected recruitment of the ligand-AR complex to a PSA enhancer region in LNCaP cells, underscoring that these differences can also affect genomic action. These findings support the notion that nongenomic pathways may also provide a potential mechanism for tissue selectivity, although its clinical relevance remains unclear.

## Clinical trials and the missing comparator

Several SARMs have advanced to clinical trials, including enobosarm (also known as GTx-024, ostarine or MK-2866) (64–67), ligandrol (also known as LGD-4033 or VK5211) (68), GSK2881078 (69), and RAD140 (70). This logically followed from the preclinical work that was done on these compounds. The advancement of enobosarm, for example, was supported by preclinical studies demonstrating robust anabolic activity in a modification of the Hershberger assay, together with favorable pharmacokinetic properties identified through systematic evaluation of arylpropionamide derivatives, from which enobosarm emerged as the most promising candidate (29). These characteristics — high potency, oral bioavailability, and apparent tissue selectivity — provided the rationale for advancing enobosarm into clinical trials. Building on this preclinical foundation, subsequent clinical trials of enobosarm and other SARMs have commonly reported improvements in lean body mass (LBM), which is unsurprising given that SARMs act on the AR. A key limitation of the clinical literature to date is that these trials are placebo-controlled rather than head-to-head. Without comparisons against conventional steroidal androgens, assessing whether SARMs are equivalent — or even superior — in efficacy or safety is difficult. A brief overview of clinical trials published in the literature is provided in **Table 2**.

**Table 2:** A brief overview of published clinical SARM trials.

Authors	Compound	Subjects	Trial phase	Outcomes
Dalton et al., 2011 (64)	Enobosarm	Healthy elderly men and postmenopausal women	Phase 2	Dose-dependent increase in lean body mass and physical function.
Yi et al., 2012 (71)	OPK-88004	Healthy men	Phase 1	Not applicable
Dobs et al., 2013 (65)	Enobosarm	Male and female cancer patients	Phase 2	Increase in lean body mass
Basaria et al., 2013 (68)	LGD-4033	Healthy young men	Phase 1	Increase in lean body mass with no change in lower body strength
Bhattacharya et al., 2016 (72)	PF-06260414	Healthy men	Phase 1	Not applicable
Coss et al., 2016 (73)	Enobosarm	Healthy men	Phase 1	Not applicable
Clark et al., 2017 (69)	GSK2881078	Healthy men and postmenopausal women	Phase 1	Not applicable
Neil et al., 2018 (36)	GSK2881078	Healthy older men and women	Phase 1	Increase in lean body mass
Yuan et al., 2021 (66)	Enobosarm (in addition to pembrolizumab)	AR-positive metastatic triple-negative breast cancer patients	Phase 2	Clinical benefit rate of 25% at 16 weeks
Pencina et al., 2021 (74)	OPK-88004	Prostate cancer survivors who had undergone radical prostatectomy	Phase 2	Increase in lean body mass, decrease in fat mass, no improvement in sexual symptoms or physical performance
LoRusso et al., 2022 (70)	RAD140	ER+/HER2 metastatic breast cancer patients	Phase 1	Clinical benefit rate of 18.2% at 24 weeks
Palmieri et al., 2024 (67)	Enobosarm	AR- & ER-positive, HER2-negative advanced breast cancer patients	Phase 2	See text.

Nevertheless, cross-trial comparisons provide some insight. In one 12-week placebo-controlled trial in healthy elderly men and postmenopausal women, the highest-dose enobosarm group increased LBM by 1.3 kg and reduced fat mass (FM) by 0.6 kg compared with the placebo group (64). HDL cholesterol, sex hormone binding globulin (SHBG), and testosterone specifically in men, decreased. Although quantitative data on liver transaminases were not specified, one subject in the highest-dose enobosarm group discontinued because of elevated alanine aminotransferase (ALT). Notably, side effects relating to virilization were not discussed in the manuscript. By comparison, a 12-week course of oxandrolone, an orally bioavailable steroidal androgen, increased LBM by 3.0 kg and reduced FM by 1.9 kg in healthy elderly men (53). No adverse events attributable to oxandrolone treatment were noted with only modest changes in blood chemistry. While there are several problems with cross-trial comparisons like this, they strongly highlight the pressing need for head-to-head comparisons — otherwise, SARMs risk being nothing more than novel AR ligands lacking superiority over long-available inexpensive alternatives.

In another trial, healthy men were randomized to placebo or varying dosages of ligandrol for 3 weeks (68). Measurements, including that of LBM, were performed 1 week after the last dose. LBM increased by 1.2 kg in the highest dose group compared with placebo. Interestingly, the authors also mention “In spite of demonstrable androgenic activity, serum prostate-specific antigen did not change significantly.” Specific mention of unchanged PSA is also made in the conclusions section of the abstract, framing this as a notable positive finding. However, this overlooks clinical data from conventional androgens that show that supraphysiological dosages of testosterone enanthate (600 mg weekly) for 20 weeks do not increase PSA in healthy young (26, 75) or older men (76) either. Given that ligandrol was administered at relatively low dosages for a short period of time, the lack of PSA elevation is hardly unexpected.

The AR also plays an important role in breast cancer, hence the AR can be included in molecular breast cancer classification (77, 78). Preclinical data spawned phase 2 trials in evaluation of the AR antagonist enzalutamide as it showed potential benefit in AR-positive breast cancer cells (79). Unfortunately, these trials demonstrated disappointing results (80, 81). Enobosarm, in contrast, shows promising anti-tumor activity in AR-positive, ER-positive, HER2-negative advanced breast cancer patients (67). Indeed, historically, androgens such as testosterone propionate, methenolone and fluoxymesterone demonstrated tumor regression in up to 30% of patients with advanced disease. However, virilizing effects and the approval of tamoxifen for treatment of women with advanced breast cancer by the Food and Drug Administration in 1977 caused androgens to fall out of favor. SARMs, by contrast, may offer a promising re-emerging therapy lacking virilization for treatment of breast cancer. In a recent phase 2 trial, 29–32% of patients receiving enobosarm experienced clinical benefit (defined as complete response, partial response, or stable disease) at 24 weeks (67). However, no patient had a complete response and only a single patient out of 92 achieved an objective partial response at 24 weeks. Adverse events were monitored and serological evaluation was performed, but virilizing side effects, such as hirsutism, were not reported. This invites comparison with older studies. In a 1967 trial, women with advanced breast cancer received extremely high dosages of the steroidal nonaromatizable androgen methenolone enanthate (1200 mg weekly) or testosterone propionate (300 mg weekly) (82). Methenolone achieved objective tumor regression in 13 (48%) of 27 patients, while testosterone achieved none. The median survival was 27 months in those responding to methenolone treatment and 7–7.5 months in those who did not respond or received testosterone. Androgen side effects, however, were common: acne, hirsutism, and hoarseness occurred in 44, 52 and 76% of patients, respectively. Although such high-dose regimens are less acceptable today, these results suggest that older nonaromatizable androgens can be highly effective. Future research should assess whether lower, less virilizing doses of such nonaromatizable androgens can match SARMs like enobosarm in both efficacy and safety.

Despite over a quarter-century of research, no SARM to date has received approval by regulatory authorities such as the Food and Drug Administration or European Medicines Agency. This lack of approval notwithstanding, the purported improved safety profile of SARMs over conventional steroidal androgens has garnered the attention of both professional and recreational athletes. A recent estimate suggests that the prevalence of SARM use among athletes is 1–3% (83). Resultingly, a growing number of case reports and series have documented serious adverse effects resembling those associated with traditional androgens, most notably drug-induced liver injury (84). This hepatotoxicity is likely linked to increased activation of the AR in hepatic tissue and may be an unavoidable consequence of orally bioavailable androgens administered at sufficiently high doses (85). Moreover, the lack of long-term experience with these novel compounds raises concerns about the potential for serious, class-specific adverse effects that are unrelated to their androgenic activity. Collectively, these factors underscore the emerging risks associated with SARM misuse.

## Conclusion

Despite decades of research and development, SARMs have yet to fulfill the promise of providing truly tissue-selective effects. Much of the observed selectivity likely stems from differences in metabolism that are not unique to SARMs and had already been achieved through earlier modification of the steroid nucleus. While insights into AR structure and differential coregulator recruitment offer theoretical avenues for achieving enhanced tissue selectivity, robust evidence linking these mechanisms to meaningful clinical outcomes is still lacking. Although SARMs were conceived to outperform conventional androgens in both efficacy and safety, the lack of head-to-head trials makes it impossible to substantiate these claims. Until such data become available, SARMs should be viewed as pharmacological concepts with theoretical appeal but unproven clinical superiority.
